# Maternal modulation of paternal effects on offspring development

**DOI:** 10.1098/rspb.2018.0118

**Published:** 2018-03-07

**Authors:** Rahia Mashoodh, Ireneusz B. Habrylo, Kathryn M. Gudsnuk, Geralyn Pelle, Frances A. Champagne

**Affiliations:** 1 Department of Psychology, Columbia University, 1190 Amsterdam Avenue, Room 406 Schermerhorn Hall, New York, NY 10027, USA; 2 Department of Zoology, University of Cambridge, Downing St, Cambridge, CB2 3EJ, UK; 3 Columbia University Medical Center, 650 W 168 St, New York, NY 10032, USA; 4 Department of Psychology, University of Texas at Austin, 108 E Dean Keeton St, Austin, TX 78712, USA

**Keywords:** maternal care, paternal germline transmission, behaviour, embryo transfer

## Abstract

The paternal transmission of environmentally induced phenotypes across generations has been reported to occur following a number of qualitatively different exposures and appear to be driven, at least in part, by epigenetic factors that are inherited via the sperm. However, previous studies of paternal germline transmission have not addressed the role of mothers in the propagation of paternal effects to offspring. We hypothesized that paternal exposure to nutritional restriction would impact male mate quality and subsequent maternal reproductive investment with consequences for the transmission of paternal germline effects. In the current report, using embryo transfer in mice, we demonstrate that sperm factors in adult food restricted males can influence growth rate, hypothalamic gene expression and behaviour in female offspring. However, under natural mating conditions females mated with food restricted males show increased pre- and postnatal care, and phenotypic outcomes observed during embryo transfer conditions are absent or reversed. We demonstrate that these compensatory changes in maternal investment are associated with a reduced mate preference for food restricted males and elevated gene expression within the maternal hypothalamus. Therefore, paternal experience can influence offspring development via germline inheritance, but mothers can serve as a modulating factor in determining the impact of paternal influences on offspring development.

## 1. Background

The paternal transmission of environmentally induced phenotypes across generations has been reported to occur following *in utero* endocrine disruptor exposure [1], postnatal stress [2] and dietary changes [3–5]. The biological mechanisms of these effects have been of particular interest as these effects have been demonstrated to occur in non-monogamous species where there is limited contact between male sires and offspring. It has been proposed that inherited epigenetic changes, transmitted through germ cells, may account for this phenomenon. Variation in paternal experiences (i.e. toxin exposure, nutrition, stress) are associated with changes in DNA methylation, histone modifications and small RNAs in paternal sperm [2–4,6,7], epigenetic marks that can be transmitted to the embryo and presumably withstand epigenetic reprogramming in the zygote [8,9].

However, evidence suggestive of a germline epigenetic pathway in mediating paternal effects has been predominantly correlational and does not establish epigenetic variation in the sperm as the exclusive mechanism responsible for altering offspring development. The germline inheritance hypothesis also fails to account for the interplay between maternal and paternal effects, which occur in varying degrees in non-monogamous mammalian species in response to prevailing mating conditions. Within the study of behavioural ecology, it has been established that females regulate the level of parental care (pre- and postnatal) provided to offspring in response to mate quality [10–12]. This regulation can take the form of ‘differential allocation', resulting in increased investment toward offspring sired by attractive/high-quality males or ‘reproductive compensation', resulting in increased investment in the offspring of unattractive/low-quality males [12,13]. We have previously demonstrated differential allocation in female mice toward the offspring of socially enriched versus socially deprived males [14]. Moreover, paternally induced changes in maternal investment have been demonstrated to impact the fitness of offspring (i.e. body size) [10]. Thus, the observed ‘inheritance' of paternal effects may actually be phenotypes that are recapitulated through indirect post-fertilization maternal effects.

In the current study, we sought to elucidate the independent contribution of maternal effects versus germline inheritance for the transmission of paternal experience towards offspring phenotype. Using natural matings and embryo transfer to generate offspring sired by a food restricted or control fed males, we hypothesized that paternal exposure to nutritional restriction would impact male mate quality and subsequent maternal reproductive investment with consequences for the transmission of paternal germline effects.

## 2. Material and methods

### (a) Adult food restriction

Adult male C57BL/6 mice (6–8 weeks of age from Jackson Laboratories, USA) housed four per cage in Plexiglas cages were food restricted (FR) for 3 weeks. During this period, mice were fed to maintain 80%–85% of their initial body weight. Feeding occurred daily at unpredictable times (between 12 and 22 h of last feeding) with varying feeding durations and quantities to increase the potency of the stress and limit potential for adaptation. Control (CF) mice were weighed daily but given ad libitum access to food. All procedures were performed with the approval of the Institutional Animal Care and Use Committee (IACUC) at Columbia University. Subsets of food restricted (FR) mice underwent behavioural testing for anxiety- and depression-like behaviour (novel open-field and forced-swim test; *N* = 10 per group per test). Males (CF and FR) that were not behaviourally tested were used in subsequent mating experiments (sample sizes described further below).

### (b) Maternal investment

Immediately following the 3-week food restriction period a single male was placed in a mating group with two adult C57BL/6 female mice for approximately 2 weeks (30 mating pairs per condition). After the mating period, males were removed and once females reached late pregnancy they were separated and singly housed for the remainder of the experiment (resulting in *N* = 54–59 successful pregnancies per group).

#### (i) Prenatal maternal investment

As a proxy measure for prenatal investment (e.g. food consumption during gestation), pregnant female mice were weighed daily across gestation. Percentage weight gain for each gestational day was calculated by subtracting current weight from initial weight and dividing by initial weight and multiplied by 100.

#### (ii) Postnatal maternal investment

Following parturition, dams were observed to determine variation in postnatal maternal behaviours. The procedure for assessing maternal behaviour in mice has been described previously [15]. Each dam was observed for four 1 h periods per day (20 focal observations per hour) by an observer blind to paternal condition from postnatal (PN) days 1–6 (with PN0 being the day of birth). The frequency of the following behaviours was recorded: mother in contact with pups, mother in nursing posture over pups and mother licking and grooming any pups (*N* = 21–30 per group). Frequency (%) of maternal behaviour was calculated as the number of observations in which a behaviour was observed divided by the total number of observations (460) and multiplied by 100.

#### (iii) Maternal gene expression analysis

A subset of pregnant females was sacrificed either during late gestation (2–3 days before birth) or on PN1 (*N* = 5–6 per group) for gene expression analysis using quantitative real-time PCR. Gene targets analysed consisted of oestrogen receptor alpha (*Esr1*), paternally expressed gene 3 (*Peg3*) and mesoderm-specific transcript (*Mest*). Previous studies have reported that deletion of *Mest* or *Peg3* reduces prenatal food intake and gestational weight gain and that *Esr1*, *Mest* or *Peg3* deletion results in disruption to postnatal maternal behaviour during the postnatal period [16–18].

### (c) Female olfactory discrimination of CF versus FR males

We tested females' ability to distinguish between male odours using a habituation–dishabituation task [19–20] and a male urine preference test [19,21]. See electronic supplementary material for a description of behavioural methods.

### (d) Embryo transfer and natural mating

We used embryo transfer as a strategy for dissociating paternal nutritional effects acting via sperm/germline-associated factors and those effects that may also involve mating-associated changes in the mothers. Embryo transfer, as opposed to *in vitro* fertilization, has been shown to induce fewer disruptions to gene expression and development [22,23]. Donor females (28–30 day old C57BL/6; *N* = 46 per group) were superovulated with an intraperitoneal (IP) injection of five IU pregnant mare serum (PMSG; EMD Chemicals), followed 47 h later with a 5 IU IP injection of human chorionic gonadotropin (hCG; Sigma). We used half the recommended dose of hormone for superovulation to minimize any effects that superovulation itself could have on offspring [22]. Superovulated donor females were mated with either FR or CF males; *N* = 25 per group). Following fertilization, 20–25 early-stage embryos (12–16 h post coitum, one cell embryo) were collected, pooled and implanted in the oviduct of pseudopregnant surrogate females (B6CBAF1 strain, all mated with vasectomized CF males; Jackson Labs). Surrogate females of this strain were used because of the limited success of C57BL/6 females as surrogate mothers. We, therefore, chose a strain that was genetically close to C57BL/6 mice (F1 hybrids derived from C57BL/6 mothers). To avoid the effects of long-term culture on embryos [23], embryos were implanted within 1.5 h of being collected. Following surgery, with the exception of daily weighing, mice were left undisturbed throughout gestation. FR or CF males (same as those used to generate embryos for the embryo transfer experiments) were mated naturally with 6–8-week-old adult C57BL/6 females (two females per male) for 2 weeks to generate adult offspring for the natural mating (NM) condition.

These experiments produced four groups of females (and offspring; *N* = 13–15 successful litters per condition): (i) naturally mated with a CF male (NM-CF), (ii) naturally mated with a FR male (NM-FR), (iii) embryo-transferred CF embryo (ET-CF) and (iv) embryo-transferred FR embryo (ET-FR). See figure 2*a* and electronic supplementary material, figure S1 for a schematic outlining how each group was derived.

### (e) Offspring measures

At birth, pups were weighed and counted but otherwise left undisturbed during the postnatal period. All litters were observed from PN1–6 to determine postnatal frequency of maternal care (licking/grooming, nursing, total contact). Following the final maternal observation on PN6, litters were weighed and counted but otherwise left undisturbed until weaning (PN28). At weaning, individual pups were weighed and placed into same-sex groups of four. From each litter, a maximum of two male and two female offspring were selected for behavioural testing to measure cognitive and anxiety/depression-like behaviours. Male and female offspring from the four conditions (*N* = 15 per group per sex) underwent a behavioural test battery starting on approximately PN55 over a 4-week period. Testing occurred in the following order: (1) open-field [26], (2) novel-object recognition [27], (3) forced-swim test [28] and (4) sucrose preference test [29] (see electronic supplementary material for full description of behavioural methods). Adult weights were also measured to examine growth trajectories across development. Approximately 2 weeks after the last behavioural test (sucrose preference) mice were sacrificed by rapid decapitation and brains were extracted, flash-frozen in chilled isopentane and stored at −80°C until homogenization. The hypothalamus was dissected on dry ice from partially thawed tissue and used for subsequent gene expression analyses. Genes were chosen on the basis of their involvement in HPA function (corticotropin-releasing factor, *Crf*) and brain function/plasticity (brain-derived neurotrophic factor, total *Bdnf* [24,25]. See electronic supplementary material, figure S1 for a detailed experimental outline.

### (f) Quantitative real-time PCR analysis

RNA was isolated from the hypothalamus (dams and offspring) using the AllPrep DNA/RNA Mini Kit (Qiagen) and reverse transcribed to cDNA using the SuperScript III First-Strand Synthesis System for RT-PCR applications (Invitrogen). RNA quality was determined to be within accepted parameters using a NanoDrop spectrophotometer. Quantitative RT-PCR was performed with 1 µl of cDNA using an ABI 7500 Fast Thermal Cycler and the Fast SYBR Green Master Mix reagent (Applied Biosystems). All primer probes (Sigma-Aldrich; see electronic supplementary material, table S1) were designed to span exon boundaries ensuring amplification of only mRNA. For each gene, CT values were normalized to cyclophillin A (endogenous control, [30]. Relative expression values were obtained by the ΔΔCT method [31].

### (g) Statistical analyses

See electronic supplementary material for full description of the statistical approaches used in the analyses.

## 3. Results

We confirmed that exposure to chronic FR results in behavioural indices of increased anxiety and depression in FR compared with CF males. The effects of FR on anxiety- and depression-like behaviour were tested immediately after the 3-week FR period. FR mice spent less time in the centre area of a novel open-field (*t*_18_ = 2.70, *p* = 0.02; electronic supplementary material, figure S2A). These differences were not due to general changes in locomotor activity resulting from FR as CF and FR mice showed no differences in total distance travelled during the 10-min test (*t*_18_ = 0.12, *p* = 0.90). Further, FR and CF showed no significant differences in the amount of fecal boli deposited during the test (*t*_18_ = 0.14, *p* = 0.89). In the forced-swim test, FR males spent more time swimming during the last 4 min of the 6 min test (*t*_18_ = 2.67, *p* = 0.02; electronic supplementary material, figure S2B) and an increased latency to passive behaviours (*t*_18_ = 2.09, *p* = 0.05; electronic supplementary material, figure S2C). This was true even after accounting for body weight differences between FR and CF males.

### (a) Female maternal investment and hypothalamic gene expression is predicted by male nutritional experience

**Figure 1.**
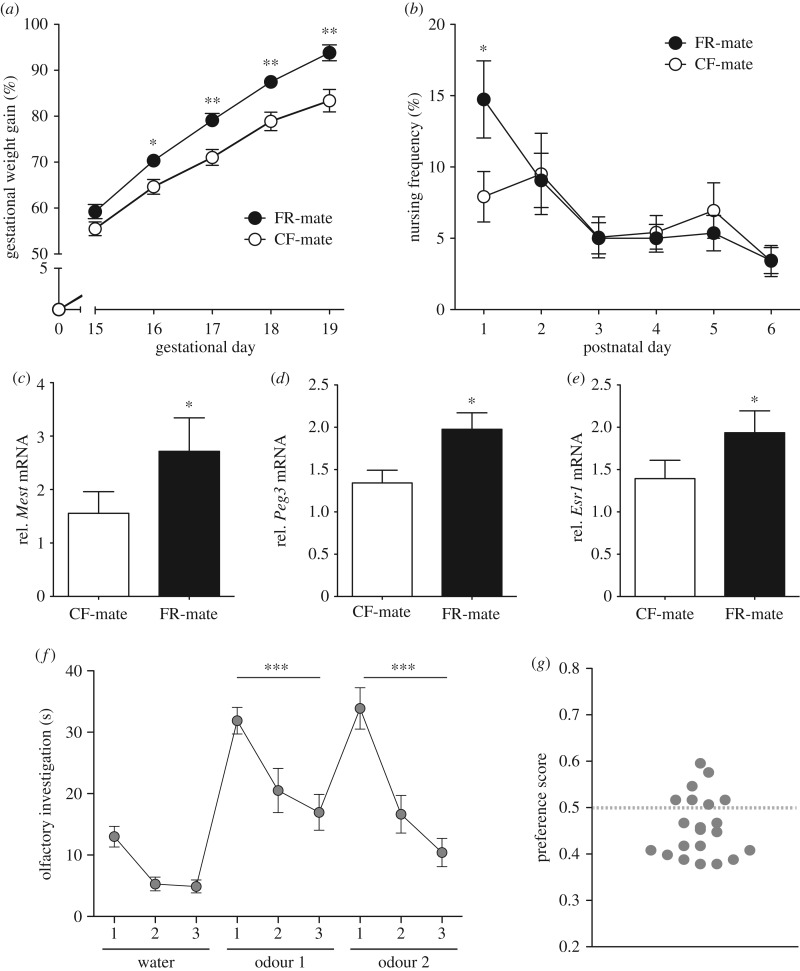
Female response to male food restriction. (*a*) Females mated with food-restricted males (FR-mated; *N* = 59) gained significantly more weight during the gestational period compared with control fed-mated females (CF-mated; *N* = 54). (*b*) FR-mated females (*N* = 21) displayed a higher frequency (%; see Material and methods) of pup nursing on postnatal day 1 compared with CF-mated females (*N* = 30). (*c*) FR-mated females have elevated levels of hypothalamic Mest mRNA during late gestation and (*d*) elevated levels of hypothalamic Peg3 and (*e*) Esr1 on postpartum day 1 compared with CF-mated females (*N* = 6 per group). (*f*) In a habituation–dishabituation task, females are able to distinguish between urine odours of FR and CF males (*N* = 20). Subsequent presentations of the same male urine odour type resulted in reduced olfactory investigation over time with a dishabituation response (increased investigation) observed following the presentation of a new odour type. (*g*) Preference scores during the urine preference task. More females preferred CF (preference score less than 0.45) than FR males (*N* = 20). *#p* < 0.10, *p < 0.05, ***p < 0.001.

Females mated with FR males exhibited increased weight gain across gestation (particularly in the last four gestational days; *t*_49_ = 6.34, *p* < 0.001) and increased pup nursing on postnatal day 1 (PN1; *t*_49_ = −2.46, *p* = 0.02; figure 1*a–b*; see electronic supplementary material for extended analyses). These effects persisted after controlling for all paternally induced changes in litter size or litter weight. Thus, gestational weight gain was independent of the effects of paternal FR on offspring growth. Moreover, these increases in maternal investment were associated with elevated levels of gene expression of *Peg3* (*t*_10_ = −2.45, *p* = 0.03; figure 1*d*) and *Esr1* (*t*_9_ = −2.43, *p* = 0.04; figure 1*e*) in the hypothalamus of lactating females*.* There was a marginally significant increase in *Mest* in the maternal hypothalamus during gestation associated with paternal FR (*t*_12_ = −1.68, *p* = 0.08; figure 1*c*). Our data suggest that FR matings induce increased maternal investment.

### (b) Female olfactory discrimination and mate preference for CF versus FR males

Females were observed to show both habitation and dishabituation to CF and FR odours indicating the capacity to discriminate between males of a CF compared with FR phenotype (effect of repeated presentation: *t*_116_ = −6.58, *p* < 0.001; figure 1*f*). Within this sensory discrimination task, we also observed an overall reduction in investigation time for FR male urine odours compared with CF odours (*t*_2_ = −2.55, *p* = 0.01). Means (±s.e.m.; in seconds) for investigation time for FR versus CF odours were 24.75 (±1.97) and 18.68 (±1.95), respectively. This effect was also observed in the three-chamber choice task, where virgin females in oestrus showed a preference for CF urine odours over FR urine odours (*χ*^2^(2, *n* = 20) = 8.50, *p* < 0.001; figure 1*g*), confirming that the FR phenotype is perceived as low quality/less attractive.

### (c) Variation in maternal reproductive investment is absent in the embryo transfer condition

**Figure 2.**
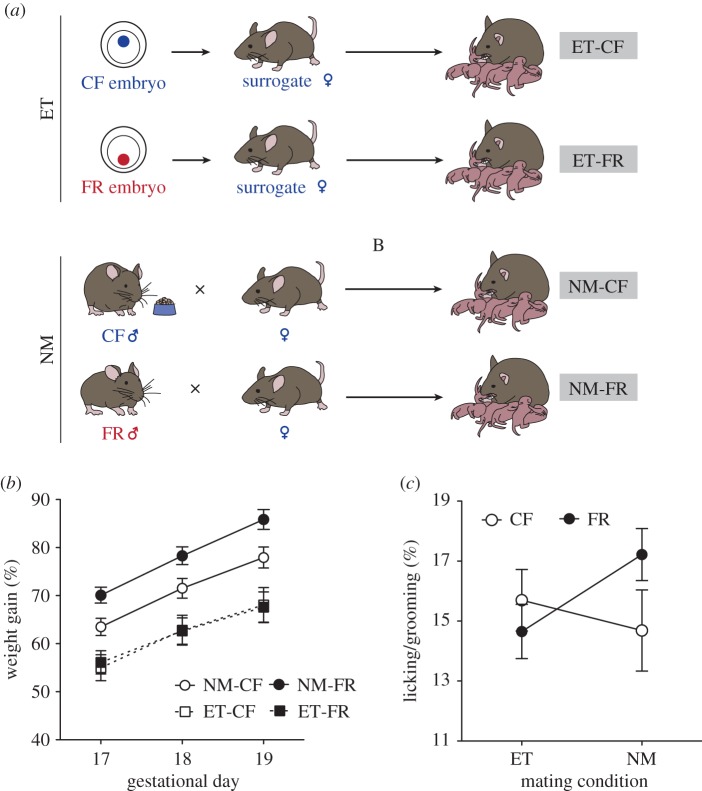
Maternal investment as a function of embryo transfer and paternal FR. (*a*) In the embryo transfer (ET) condition, surrogate females were mated with vasectomized control fed (CF) males and then implanted with embryos from food restricted (FR) or CF males (generated through matings with superovulated females) to generate ET-CF and ET-FR groups. NM-CF and NM-FR groups were generated through natural matings of females with either CF or FR males, respectively (*N* = 15 per group). (*b*) Gestational weight gain increases in response to paternal FR under NM conditions but not in embryo-transferred mothers. (*c*) Pup licking/grooming is increased on PN1 in response to paternal FR in naturally mated but not embryo-transferred females. **p < 0.01, ^#^*p* < 0.05.

FR-induced changes in prenatal weight gain and postnatal maternal behaviours were only present in NM females. Significant increases in weight gain across gestation were observed in females carrying FR pups that were conceived through NM (*t*_1050_ = 4.23, *p* < 0.001; figure 2*b*) but not embryo transfer conditions (*t*_671_ = 0.95, *p* = 0.33). There were marginally significant effects of paternal FR on frequency of postnatal maternal licking behaviour (*t*_60_ = 1.68, *p* = 0.09), which was primarily driven by differences between NM-FR and NM-CF groups (figure 2*c*).

### (d) Dissociating maternal and paternal influences in FR-associated outcomes in offspring

#### (i) Effect of FR on offspring growth

We compared growth, behaviour, and hypothalamic gene expression in CF and FR offspring generated using ET (ET-CF and ET-FR) and offspring sired through natural matings (NM-CF and NM-FR; figure 2*a*). Using the NM versus ET breeding design, we first measured the impact of paternal FR on offspring growth rates (see electronic supplementary material for detailed analyses). At weaning (PN28), ET-FR male offspring were smaller in body weight than ET-CF male offspring (*t*_111_ = −2.52, *p* = 0.01). In adulthood (PN80), body weights were reduced in male (*t*_48_ = −2.30, *p* = 0.03) and marginally in female (*t*_51_ = −1.67, *p* = 0.10) offspring in response to FR within the ET condition. No effects of FR on body weight were observed within the NM condition (electronic supplementary material, table S3).

#### (ii) Effect of FR on offspring behaviour

**Figure 3.**
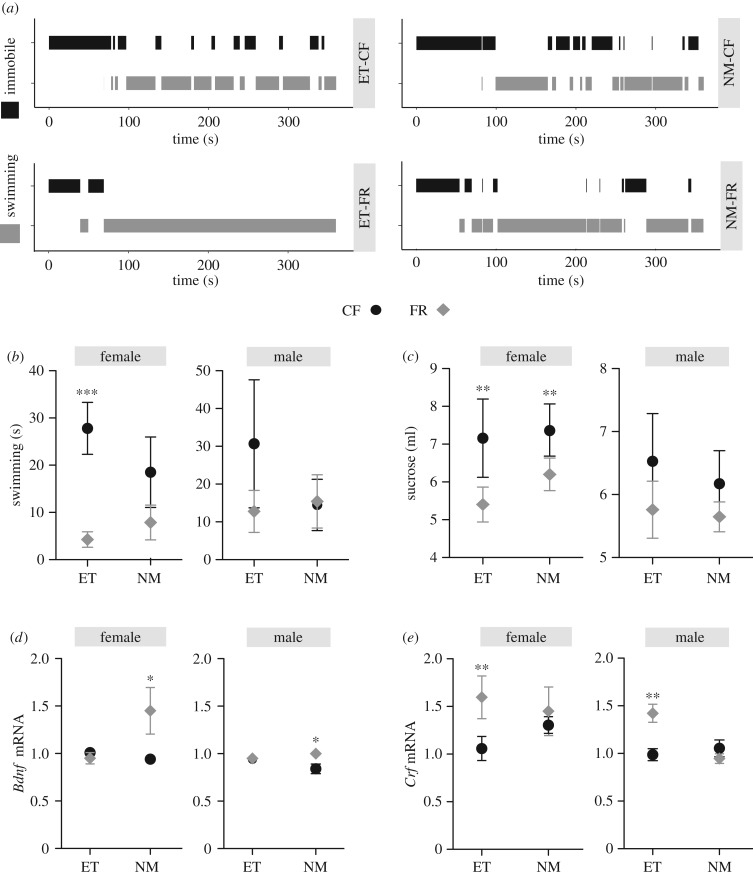
Depression-like phenotypes of offspring sired by food restricted fathers. (*a*) Representative activity plots of female offspring behaviour during the forced-swim test from all four rearing conditions. (*b*) Female offspring of FR fathers (diamonds) compared with CF (circles) had reduced active swimming time in the last 4 min of a forced-swim test only when sired through ET. No effects of FR were found in males in either mating condition. (*c*) Female and male offspring of FR fathers (diamonds) consumed less sucrose compared with offspring of CF (circles) regardless of mating condition. (*d*) Offspring of FR fathers (diamonds) had higher Bdnf mRNA levels than CF offspring (circles) only if born under NM conditions. (*e*) Crf mRNA levels were elevated in offspring of FR (diamonds) compared with CF fathers (circles) if sired through ET (*N* = 15 per group). *p < 0.05, **p < 0.01, ***p < 0.001.

**Figure 4.**
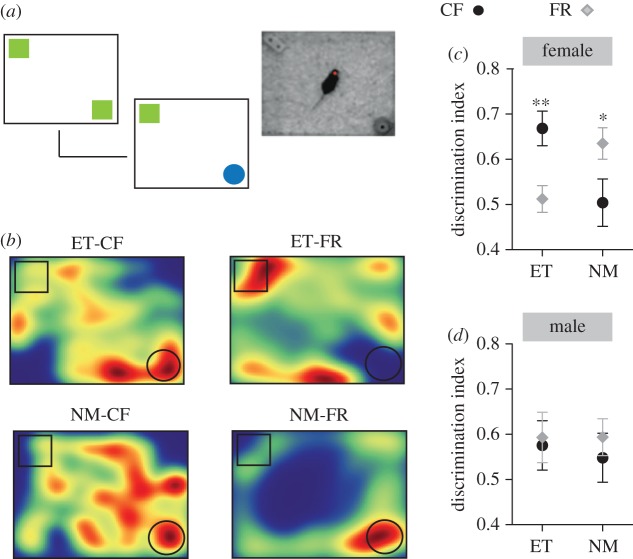
Cognitive behaviour of offspring sired by food restricted fathers. (*a*) The novel-object recognition task involved presentation of two identical objects followed by a 30 min delay after which the objects were replaced with a familiar (same object) versus novel object to test for memory of the objects. (*b*) Representative heat maps indicating the proportion of time spent in the test arena for female offspring that were born to control (CF) and food-restricted (FR) fathers under embryo transfer (ET) and NM conditions, red indicating most time spent and blue indicating least. (*c*) Female offspring of FR fathers (diamonds), compared with offspring of CF fathers (circles), show a lower discrimination index under ET conditions but higher discrimination index under NM conditions (*N* = 15 per group). (*d*) No differences in discrimination index were found in male offspring (*N* = 15 per group). *p < 0.05, **p < 0.01.

There were no effects of paternal FR on general locomotor activity or time spent exploring the centre of the open field (see electronic supplementary material, table S4). Males born to FR fathers in the embryo transfer condition exhibited a marginally shorter latency to enter the centre area (*t*_112_ = −1.87, *p* = 0.06). Depression-like behaviours were also assessed in offspring of CF and FR males. In the forced-swim test, paternal FR reduced the total duration of active swimming in female offspring generated through ET (*t*_27_ = −3.97, *p* < 0.001; figure 3*b*). Assessment of sucrose intake also revealed FR-induced increases in depression-like behaviours. In female offspring (and to a lesser degree in males) generated through both NM and ET, paternal FR was associated with reduced sucrose consumption (after controlling for overall intake; *t*_54_ = −2.62, *p* = 0.01; figure 3*c*). Within the novel-object recognition task, the discrimination index was found to be significantly higher in NM-FR compared with NM-CF female offspring (*t*_28_ = 2.08, *p* = 0.04) but reduced in ET-FR compared with ET-CF female offspring (*t*_28_ = −3.22, *p* < 0.01; figure 4*c–d*).

#### (iii) Effect of FR on offspring hypothalamic gene expression

Both male and female NM-FR offspring had elevated levels of *Bdnf* expression compared with NM-CF offspring (*t*_27_ = 2.87, *p* < 0.001; figure 3*d*). This effect on *Bdnf* expression was not observed under ET conditions (*t*_28_ = −0.92, *p* = 0.32). By contrast, both male and female ET-FR offspring had elevated levels of *Crf* expression compared with ET-CF offspring (*t*_30_ = 3.53, *p* < 0.001), an effect not observed under NM conditions (*t*_29_ = 0.01, *p* = 0.99; figure 3*e*).

## 4. Discussion

Our findings indicate that the effects of paternal food restriction vary depending on the sex of offspring and mating conditions. Offspring born to food-restricted fathers and derived through embryo transfer show growth deficits, impairments in recognition memory and behaviours indicative of learned-helplessness and anhedonia. These changes were associated with increased *Crf* expression in the hypothalamus of offspring indicative of heightened stress reactivity [25,32]. Importantly, these effects were found primarily in females suggestive of an increased sensitivity of female offspring to paternal food restriction cues transmitted through the germline. In contrast, when offspring were conceived through NM, FR female offspring showed improved recognition memory and no differences in forced-swim behaviour compared with CF offspring. The only similarity in offspring outcomes as a function of mating condition was that, like embryo-transferred FR female offspring, FR offspring conceived through NM also displayed reduced sucrose intake. We propose that the differences in transmission direction and magnitude of paternal food restriction effects are due to increases in maternal investment observed in females following NM with FR males.

### (a) Germline effects of paternal food restriction

Chronic FR is a psychological and physiological stressor that draws parallels to the type of stress experienced in response to famine or poverty. The experience of chronic stress associated with dietary restriction, as shown in both humans and animal models, has broad repercussions for metabolic, cognitive, emotional and motivational domains of behaviour with consequences for offspring development [33,34]. For example, archival data from Sweden indicate that food availability (during famine) of grandfathers is associated with the risk of diabetes and cardiovascular disease as well as mortality in grandsons [5,35]. The growth, memory- and stress-related deficits in female offspring born to FR fathers through embryo transfer (ET-FR) is indicative of paternal transmission of stress-related phenotypes. These findings are consistent with the accumulating evidence that, in addition to maternal depression, history of stress in fathers may contribute to similar outcomes in offspring [2,5,6,35–37]. Our data suggest that FR-induced variation in the germline has the capacity to influence stress-related behavioural outcomes.

The underlying mechanism of paternal effects that occur in the absence of postnatal father–offspring contact is assumed to involve epigenetic changes within the germline (e.g. piRNA or other small RNAs, DNA methylation) [7,38]. Data from our embryo-transfer condition support a direct germline route for paternal influences and are consistent with the partial transmission of paternal effects after artificial reproduction observed in previous studies [2,6,36,38]. However, there are caveats in the use of artificial reproductive techniques such as of IVF and embryo transfer in demonstrating the transmission of acquired epigenetic marks. Several features of the methods used within artificial reproductive techniques may themselves alter epigenetic reprogramming. For example, stimulation of oocyte production using gonadotrophins (superovulation) with high levels of hormone is routinely used to improve efficiency in human-assisted reproduction and in the generation of genetically modified laboratory rodents. Comparison of DNA methylation patterns in embryos derived from superovulated versus non-superovulated females indicates that this procedure may induce abnormal DNA methylation patterns [22,39]. IVF has been similarly found to induce changes in embryonic DNA methylation patterns depending on the type of culture media used for the incubation of sperm and oocytes [40]. Moreover, the duration of time that embryos spend in culture prior to implantation also has effects on anxiety-like behaviour in adult embryo-transferred mice [23]. These abnormalities in DNA methylation may account for reports of an increased incidence of imprinting disorders (such as Angelman and Prader–Willi syndrome) in individuals conceived through these procedures [41]. However, we mitigated these issues by using reduced levels of hormone to stimulate ovulation, embryo transfer (as opposed to IVF) to avoid external fertilization in a culture medium, and minimized the time embryos spent outside the body by transferring embryos immediately. These methodological issues will be critical to consider in the interpretation of previous and future studies of paternal and maternal effects on offspring development.

### (b) Role of paternally induced maternal effects

Under NM conditions, female offspring of FR fathers exhibited improved cognitive performance and were not observed to have altered growth trajectories or depression-like behaviour in the forced-swim test compared with offspring of CF fathers. The reduced sucrose preference observed, though typically interpreted as an indication of anhedonia, is probably an appetitive phenotype in the context of increased levels of hypothalamic *Bdnf* expression in FR offspring. *Bdnf* is heavily expressed in energy balance centres within the hypothalamus and loss of *Bdnf* in these regions has been shown to induce hyperphagia and obesity in mice [42,43]. Moreover, FR offspring derived through NM showed no indices of stress or anxiety. Therefore, reductions in sucrose intake in FR offspring may be an adaptive response to paternal metabolic phenotype.

The divergence in phenotypic outcomes resulting from paternal FR under embryo transfer versus NM conditions probably results from the differential allocation of maternal resources received by these two groups. Females that mated with FR males showed increased levels of gestational weight gain and postnatal maternal behaviour. Moreover, these behavioural changes were associated with increases in hypothalamic *Mest* mRNA during late gestation and *Peg3* during the first postnatal day of females mated with FR males—genes that have been shown to regulate prenatal food intake and maternal behaviour [16–18,44,45]. Elevated *Esr1* expression in the maternal hypothalamus observed during lactation in FR-mated females probably has consequences for multiple neural systems regulating maternal behaviour including oxytocinergic and dopamine pathways [46,47].

We suggest that these paternally induced maternal effects serve as a compensatory response. The notion of paternally induced maternal effects has remained largely unexplored in laboratory animals but may explain why paternal phenotype can result in paradoxical effects on offspring [48,49]. Changes in postnatal maternal care can shape the neural systems underlying stress, anxiety, cognition and brain plasticity through epigenetic mechanisms that result in stable levels of gene expression throughout life [50]. The nutritional environment during fetal development has likewise been shown to be critically important for growth, metabolism, brain development and behaviour via epigenetic mechanisms [51]. Thus, while the embryo-transfer condition in the current study suggests that paternal FR impedes offspring growth and impairs cognitive/behavioural functioning, increased food-intake and maternal behaviour observed in FR-mated females may serve to buffer offspring from these effects by overriding paternal influences on gene expression via maternally mediated epigenetic mechanisms.

### (c) Sources of paternally induced maternal effects

A critical question that emerges when considering the compensatory influence of mothers on germline paternal effects is the route through which fathers can trigger altered maternal investment. Following implantation, embryonic and maternal physiology become intricately coordinated and the continued growth and development of the embryo/fetus are dependent on the release of growth factors and hormones from the fetoplacental unit [52]. Paternally expressed genes, which are susceptible to epigenetic modification, are highly expressed in the placenta, critical for fetal growth (e.g. *Mest*), and can regulate postnatal mother–infant interactions (e.g. *Peg3* and *Gnasxl*). Further, in rodents, it has been demonstrated that during the postnatal period, offspring traits such as locomotor activity, suckling ability, and ultrasound production enable pups to regulate the levels of maternal care they receive from the dam, leading to altered developmental trajectories [16,53]. Therefore, paternal epigenetic variation present within imprinted genes that is transmitted to offspring could lead to shifts in the level of prenatal food intake and/or priming of maternal behaviours through either effects on placental function or on pup behaviour. However, our data indicate that paternal germline alterations that affect the placenta or pup directly are unlikely to account for the increased maternal investment observed. Increased maternal investment in FR offspring was not observed following embryo transfer, a condition in which female mates do not have mating experience with FR males. Moreover, females can clearly distinguish between FR and CF males and show a reduced preference for FR males. Overall, these data suggest that reproductive compensation occurs as a consequence of the perceived mate quality of food restricted males. Studies of mate preference have typically focused on genetic features of males that would potentially impact offspring viability (e.g. MHC complexes) [54,55]. However, there is increasing evidence that females make preference distinctions based on males' prior experience (e.g. *in utero* undernutrition, vinclozalin and parasite exposure) [56–58]. In the case of FR-mated females, this lack of preference, combined with the constraints on mating opportunities available, may lead to increased maternal investment and altered offspring developmental trajectories.

### (d) Sex-Specific effects of paternal food restriction

Consistent with previous studies examining the impact of fathers, we find that female offspring are most sensitive to the effects of paternal food restriction. Studies of the impact of advanced paternal age on autism risk indicate that older fathers are more likely to have daughters with autism [59]. Daughters with a history of paternal alcoholism are more sensitive to the effects of benzodiazepenes [60]. A similar sex-specificity has been found in laboratory studies of paternal effects. Paternal stress exposure, with stress occurring either in early life or during juvenile development, results in increased emotional reactivity and impaired social behaviour in female offspring. Interestingly, though paternal stress does not impact behavioural phenotypes in male offspring, males are capable of transmitting the phenotype to their offspring suggesting that males act as ‘carriers' of the epigenetic mark [61,62]. Possible explanations for the sex-specificity of paternal effects may include sex-chromosome-linked paternal epigenetic variation, contribution of *in utero* hormones or sex differences in the timing of epigenetic reprogramming events that render females more sensitive to altered paternal germline epigenetic variation [7].

### (e) Concluding remarks

Advances in our understanding of the transmission of epigenetic variation across generations has generated increased interest in the mechanisms of paternal germline effects. Though our data provide evidence of paternal germline effects, the occurrence of paternally induced compensatory maternal responses that we have observed suggest a highly dynamic interplay between mothers and fathers in shaping offspring outcomes. The product of these interactions can have implications for the direction/magnitude of offspring outcomes as well as the degree of penetrance of paternal experience (i.e. the degree and number of subsequent generations that can be affected). Therefore, determining the effect of the male germline requires a thorough understanding and/or control of maternal effects, and considers the dynamics of mating, the physical and hormonal exposure of oocyte and embryos, and the environmental conditions *in utero* and postnatal that reflect the interactions between offspring and mother. Our findings argue for a more inclusive notion of inheritance, incorporating genetics, epigenetics and the social context, when predicting the transgenerational impact of parental experiences.

## Supplementary Material

Supplementary materials

## Supplementary Material

Supplementary data - Maternal

## Supplementary Material

Supplementary data - Offspring

## Data accessibility

All data used in analyses and figures are included in the electronic supplementary material.
