# Supplementary material for: Maternal modulation of paternal effects on offspring development
Source: Proc Biol Sci. 2018 Mar 7;285(1874):20180118. doi: 10.1098/rspb.2018.0118 (PMC5879637; doi:10.1098/rspb.2018.0118)
Supplement: Supplementary materials [file rspb20180118supp1.doc]

Supplementary Materials

**BEHAVIOURAL METHODS**

*Habituation-Dishabituation*: Oestrus-stage females (selected on the basis of vaginal cytology the morning prior to testing; N=20) were placed in a new home cage and permitted to habituate for 30 min to the testing room. Tests involved nine sequential presentations of olfactory stimuli (a 2cm square of filter paper saturated with 30μl of urine or water): three of water, followed by three of one male urine type (CF or FR, each pooled from 2-3 males in that condition), and finally three of the other male urine type (see (19). Olfactory stimuli were placed on top of the cage lid, and each stimulus presentation lasted two minutes with a 1-min interval between presentations. The order of urine type presentation was counterbalanced across subjects. Subjects were scored as being engaged in investigation when they were sniffing the air directly beneath the stimuli (20).

*Male Urine Preference Test*: Oestrus-stage female mice were tested for their preference of urine odour of CF *vs.* FR male mice using a three-chambered apparatus (19). The apparatus was a 70cm x 20cm x 20cm Plexiglas arena with 7.5cm x 3cm openings in each equally-sized partition allowing subjects to move between chambers. The subject was placed in the arena and allowed to habituate for 30 min. At the end of the habituation period, the partitions were closed, confining the subject to the central chamber (5 min), and two different urine-primed olfactory stimuli (20μl of urine pooled from 4-5 males from that condition) were placed in the end chambers and covered with raised metal grilles that prevented physical contact. The partitions were then removed allowing the subject mouse to explore all three chambers. The time the subject female spent sniffing each stimulus over the following 10 min was recorded and scored using the automated tracking software (AnyMaze, Stoetling). Preference for FR males was computed by taking the time spent investigating the FR male urine odour divided by the time spent exploring either male urine odour (CF plus FR). Therefore, preferences over 0.55 are indicative of a greater proportion of time spent investigating (and therefore, preference for) the FR male urine odour. Conversely, females with a preference score of less than 0.45 were considered to have a preference for CF male urine odours (21).

*Open Field Test*: The open field apparatus used was a 60 x 60 x 40cm Plexiglas box with black walls and a white floor. On the day of testing, the mouse was removed from its home cage and placed directly into one corner of the open field. After a 10-min session, the mouse was returned to its home cage. All testing was conducted under red lighting conditions. Behaviours were video recorded. Anxiety-like and exploratory behaviours (26) scored using Ethovision (Noldus) included: (1) centre area exploration, defined as the time spent in the inner (30 x 30cm) area, (2) latency to enter the centre, and (3) distance travelled.

*Novel Object Recognition*: To assess recognition memory (27), mice were placed in a novel testing arena (28 x 28 x 30cm) for a 15 min habituation period. The following day, subjects were placed in the testing arena and allowed to explore the arena for 15 min. Following this habituation period the subject mouse was presented with two identical objects (plastic blocks or clay cylinder) that were placed on 2 opposite corners of the cage for a 15 min exploration session. Mice were then removed from the apparatus and returned to their home cage for 30 min. During this time one of the objects was replaced with a novel object. Mice were returned to the testing area for a 15 min session to explore the two objects. All trials were video recorded and scored for object exploration using AnyMaze (Stoetling). The discrimination index was calculated by diving the time spent exploring the novel object by the total amount of time spent exploring either object. Impaired recognition memory is associated with reduced exploration of a novel object (lower discrimination index) (27). Within the 8 groups of mice tested in this study, 6 groups performed the task with a discrimination index greater than 50% (using one sample t-tests, all adjusted p-values less than 0.05)

*Forced-Swim Test*: Depression-like behaviour of CF and FR mice was measured during a brief forced-swim test (28). Mice were placed into a 2L glass beaker filled with water at room temperature (approximately 25 ± 2°C). All tests were video recorded for later scoring by an observer blind to experimental condition. The behaviours scored were the latency and frequency of active struggling (vigorous swimming) and immobility (passive swimming, little to no active movement).

*Sucrose Preference Test*: Preference for sucrose, indicative of a hedonic response, was measured by a free-choice two-bottle preference task (29). Each mouse to be tested was housed alone in a new home cage. On the first day of testing, mice were provided with two drinking bottles (both containing water). On the second day, one bottle was replaced with a new bottle containing a 1% sucrose solution. Water and sucrose consumption was measured every 24h for 2 days by weighing the bottles. The position of the bottles was counterbalanced across the groups and switched after 24h. Following the test period, mice were returned to their home cage.

**STATISTICAL ANALYSES**

The effect of FR on male behaviour was analysed using a two-sample t-test. Changes in prenatal weight gain were analysed using a repeated measures ANOVA with gestational day as a within-subjects measure, mating group as a between-subject variable and time spent with male during mating and litter size included as covariates. Two-sample t-tests were used to measure differences in percent weight gain between CF- and FR-mated females on specific gestational days. Maternal hypothalamic gene expression during gestation and post-partum were analysed using a two-way ANOVA with mating group and reproductive time point as between-subject variables. Fishers test of exact differences was used to determine if female preferences were shifted towards CF or FR males in the mate odour preference task. Olfactory habituation-dishabituation was analysed using a linear regression model with order of presentation, odour type and trial number as factors.

In the embryo transfer experiments, changes in maternal investment as a function of mating condition and paternal condition was analysed using a least-squares regression model with mating condition (ET or NM), paternal condition (CF or FR), and gestational day as factors of interest and litter size as a covariate. Maternal behaviour on PN1 was analysed using a two-way ANOVA with mating condition and paternal condition as factors. Offspring growth, behaviour and hypothalamic gene expression was performed using a least squares regression model with mating group and paternal condition as factors (plus any relevant covariates). We analysed sexes in separate models as there are known sex differences in paternal responses and we were more interested in the response within sexes rather than between. Models were reduced to assess the source of any significant interactions with post-hoc two-sample t-tests.

Due to the hierarchical nature (*i.e.* sometimes multiple offspring from a single mother and/or father or multiple mates for males), where appropriate the data presented in these experiments were analysed using a more stringent multi-level model where father ID, mother ID and pup ID were allowed to have a unique intercept. The only case in which a multilevel model was not used is the behavioural analyses from the embryo transfer experiments as the design (father ID of embryos is not uniform within a litter in the embryo transfer condition as embryos were pooled from approximately 12-15 males and randomly implanted). Therefore, while father ID is known for natural mating condition the true father ID is not known in the embryo transfer condition. The effects of repeated sampling were minimized by sampling 1 pup from each litter for each of the groups. When this was not possible a maximum of 2 were sampled though this was rare.

**SUPPLEMENTARY RESUTS**

***Behaviour of FR males***

The effects of FR on anxiety- and depression-like behaviour were tested immediately after the three-week FR period. FR mice spent less time in the centre area of a novel open-field (t(18)=2.70, P=0.02; **Supplemental Figure 2A**). These differences were not due to general changes in locomotor activity resulting from FR as CF and FR mice showed no differences in total distance travelled during the 10 min test (t(18)=0.12, P=0.90). Further, FR and CF showed no significant differences in the amount of faecal boli deposited during the test (t(18)=0.14, P=0.89). In the forced-swim test, FR males spent less time swimming during the last 4 min of the 6 min test (t(18)=2.67, P=0.02; **Supplemental Figure 2B**) and an decreased latency to passive behaviours (t(18)=2.09, P=0.05; **Supplemental Figure 2C**). This was true even after accounting for body weight differences between FR and CF males.

***Prenatal Weight Gain***

Females mated with FR males show a significant increase in percent weight gained across the entire gestation period (GD0-19) as compared to females that mated with CF males (t(108)=6.34, P<0.001) and this relationship is maintained after controlling for the number of pups born and the amount of time spent with the male during the mating period (**Figure 1A**). These two variables were included in the regression model as they both were shown to significantly influence the percent of weight gain for females. As expected, larger litter sizes were associated with more weight gain (t(2234)=6.36, P<0.001) though there was no significant effect of male condition (CF *vs.* FR) on litter size (t(108)=0.41, P=0.68) or litter weight after controlling for size (t(108)=-0.09, P=0.93). Further, the number of days spent with the male mate was negatively related to weight gain across gestation (t(2234)=-8.31, P<0.001). Changes in prenatal weight gain were most pronounced for the last 4 days of gestation. Increases in weight gain in females mated with FR males were not significantly different on GD15 (t(108)=1.28, P=0.20) but were on GDs 16 (t(108)=2.42, P=0.02), 17 (t(108)=3.08, P<0.001), 18 (t(108)=3.05, P<0.001) and 19 (day before birth; t(108)=3.57, P<0.001)).

***Postnatal Maternal Behaviours***

All analyses of postnatal maternal behaviours were conducted using litter size as a covariate. There were no significant differences in total frequency of maternal contact (F(1,5)=0.33, P=0.57) or frequency of licking/grooming (F(1,5)=0.53, p=0.47) across the postpartum week in females that had mated with CF *vs.* FR males. There was, however, a significant increase in frequency of nursing on PN1 (t(49)=-2.46, P=0.02) but not on subsequent postnatal days in females that mated with FR males compared to CF males (**Figure 1B**).

***Maternal Gene Expression***

Analysis of hypothalamic gene expression in the brain of females mated with FR and CF males indicated that there was a marginally significant increase in *Mest* in FR-mated *vs.* CF-mated females during gestation (t(12)=-1.68, P=0.08; **Figure 1C**). Further, there was a significant increase on PN1 of *Peg3* expression (t(10)=-2.45, P=0.03; **Figure 1D**) and *Esr1* expression (t(9)=-2.43, P=0.04; **Figure 1E**) in females mated with FR males compared to CF-mated females.

***Female Discrimination and Preference of FR Urine Odours***

Females in oestrous were able to distinguish between urine odours of FR and CF males. In a habituation-dishabituation task, subsequent presentations of the same male urine odour type resulted in reduced olfactory investigation over time (t(116)=-6.58, P<0.001; **Figure 1F**) followed by increased investigation when a new odour was presented. Further, there was a reduction in overall investigation time for FR male urine odours compared to CF odours (t(2)=-2.55, P=0.01) indicating that this urine-type was not preferred. In a subsequent odour preference task, we measured the preference of oestrous females for an FR odour by calculating a preference score (time spent exploring FR urine divided by total time spent exploring either urine odour) with preference scores of 0.55 or more indicating a preference for FR urine and scores of 0.45 or less indicating a preference for CF. In the preference task, within the first 5 min, fewer females showed a preference for FR males (χ^2^(2, N=20) = 18.50, P<0.001).

***Maternal Investment in Embryo Transfer Conditions***

The effects of paternal FR upon gestational weight gain was dependent upon mating conditions and gestational day (three-way interaction term: t(1720)=-2.91, P<0.001; **Figure S3A**). In other words, after controlling for litter size and gestational day, significant increases in weight gain across gestation were observed in females carrying FR pups that were conceived through natural mating (NM; t(1050)=4.23 , P<0.001) but not embryo transfer conditions (ET; t(671)=0.95 , P=0.33). The differences amongst NM-CF and NM-FR females (controlling for litter size) were strongest on the last few days of gestation, with the biggest differences being evident on GD16 (t(51)=2.88, P<0.001), GD17 (t(44)=3.02, P<0.001), GD18 (t(57)=2.79, P<0.001), GD19 (t(58)=2.53, P=0.01) and GD20 (t(58)=2.86, P<0.001).

In our previous studies of maternal postnatal investment as a function of paternal experience, changes in maternal behaviour were restricted to the period immediately after parturition *(*11). In the current study, maternal behaviours were only analysed on the first postnatal day. All analyses were performed controlling for litter size and litter weight. There were no significant differences in total contact between females and their litters in either paternal condition (t(60)=0.76, P=0.45) or across the mating conditions (t(60)=-0.56, P=0.58). Similarly, there were no effects of paternal condition on nursing behaviour between NM or ET females (interaction: t(60)=-0.19, P=0.85) though there was generally more nursing performed by females within the ET group (t(60)=2.10, P=0.04). There were, however, marginally significant effects of paternal FR upon maternal licking behaviour (t(60)=1.68, P=0.09), which was primarily driven by differences between NM-FR and NM-CF groups (**Fig. S3B**)

***Assessment of Offspring***

*Litter Characteristics & Offspring Growth*

In general, ET surrogate females gave birth to litters that weighed less compared to NM females (t(62)=-2.27, P=0.03), though there was a trend towards a paternal effect of FR on total litter weight (t(62)=-1.65, P=0.10); **Table S2**). This was also true of litter size (number of pups) with ET surrogates giving birth to smaller litters (t(62)=-2.09, P=0.04) but with an overall trend towards smaller litters born to FR males compared to CF males regardless of the mating conditions (t(62)=-1.76, P=0.08). There were no effects of paternal condition on litter weight in either mating condition after controlling for these differences in size (t(62)=0.32, P=0.75).

On PN6, there were no significant effects of mating condition (ET *vs.* NM; t(62)=1.56, P=0.12) or paternal condition (CF *vs.* FR; t(62)=0.43, P=0.43) on the total litter weight. When litter weight was controlled for with litter size, there was a significant interaction between paternal condition and mating condition. More specifically, paternal FR reduced litter weight (over and above the number of pups present; t(61)=2.26, P=0.03) but this was driven by a significant difference amongst litters in the NM condition (t(27)=2.27, P=0.03). At weaning (PN28) there were significant differences in body weight dependent on sex and mating condition. ET-FR male offspring were smaller in body weight than ET-CF male offspring (t(111)=-2.52, P=0.01). On PN80, body weights were reduced in both male (t(48)=-2.30, P=0.03) and marginally in female (t(51)=-1.67, P=0.10) offspring in response to FR only in the ET condition.

*Offspring Open Field Behavior*

There were no significant effects of paternal condition (t(112)=0.86, P=0.39) or maternal mating condition (t(112)=-0.75, P=0.46) on distance travelled by offspring of either sex in the open field test (**Table S4**; paternal by maternal by sex interaction: t(112)=0.28, P=0.78). Similarly, time spent in the center area of the novel open field was not affected by paternal (t(112)=-0.00, P=0.99) or maternal mating (t(112)=-0.78, P=0.45) conditions in offspring of either sex (3-way interaction term: t(112)=-0.84, P=0.40). There was, however, an effect of paternal FR upon the latency to enter the centre area that was dependent on mating condition and sex of offspring (t(112)=-1.87, P=0.06). Specifically, there was a shorter latency to enter the centre in males born to FR fathers in the embryo transfer condition.

*Novel Object Recognition*

Recognition memory as measured by a discrimination index was shown to be influenced by an interaction between paternal condition, maternal mating condition and sex (t(112)=1.98, P=0.049; **Fig. 3**). In females, the discrimination index was significantly higher in NM-FR offspring (t(28)=2.08, P=0.04) but reduced in ET-FR offspring (t(28)=-3.22, P<0.01). In contrast, there were no significant differences amongst male offspring across either maternal mating (t(56)=-0.24, P=0.81) or paternal condition (t(56)=0.01, P=0.99).

*Depression-Like Behaviour*

Paternal food restriction reduced sucrose consumption (after controlling for overall intake) over the two days of testing (t(106)=-2.80, P<0.001; **Fig. 2D**) and this effect was sex dependent. Female offspring, regardless of whether they we sired through ET or NM showed significant overall reductions in sucrose consumption if the father was FR (t(54)=-2.62, P=0.01). A similar pattern was evident in males, where there was overall a significant trend towards reduced sucrose consumption (t(51)=-1.606, P=0.12) that reached significance in ET male offspring (t(24)=-1.92, P=0.062) but remained a trend in NM male offspring (t(26)=-1.62, p=0.114; **Fig. 2D**).

Paternal FR compared to CF reduced the total duration swimming in a forced swim test when animals were sired through embryo transfer (t(55)=-1.73, P<0.01; **Fig. 2C**). These effects were primarily driven by significant reductions in active swimming in ET-FR compared to ET-CF female offspring (t(27)=-3.97, P<0.001). No differences were found amongst NM females or males in swimming behaviour. Importantly these effects were found to be true even after controlling for offspring adult body weight.

*Offspring Hypothalamic Gene Expression*

Paternal FR increased mRNA levels of *Crf* in the hypothalamus in offspring (both male and female) sired through ET (t(30)=3.53, P<0.001; **Fig. 2F**) but not NM (t(29)=0.01, P=0.99). In contrast, paternal FR resulted in reduced levels of *Bdnf* mRNA in the hypothalamus of offspring sired through NM (t(27)=2.87, P<0.001; **Fig. 2E**) but not the ET (t(28)=-0.92, P=0.32) condition.

**Figure S1** **Detailed diagram of all mice involved in generating maternal conditions for assessing paternal FR**. **(A)** Surrogate females mated with vasectomized CF males were implanted with either embryos from FR or CF males to generate ET-CF and ET-FR groups. NM-CF and NM-FR groups were generated through natural matings of females with either CF or FR males, respectively. **(B)** Experimental timeline for paternal CF/FR offspring. Following embryo transfer and natural matings, maternal investment of mothers was measured. Offspring were weaned on PN28 and behaviorally tested over a one-month period starting on PN55. Body weight measurements were taken on PN0, PN6, PN28 and PN80. Offspring were sacrificed and brains were collected on PN90.

**Figure S2 Behavior of food restricted (FR) males. (A)** FR males spent less time in the center during a 10 min open-field test compared to control fed (CF) males. **(B)** FR males spent less time swimming during a 6 min forced-swim test compared to CF males. **(C)** Latency to enter passive swimming state was lower in FR males compared to CF males. **P < 0.05*

**Table S1: Primer sequences for qPCR**

| Gene | Forward Primer (5') | Reverse Primer (3') |
| --- | --- | --- |
| *Bdnf* | CATAAGGACGCGGACTTGTACA | AGACATGTTTGCGGCATCCA |
| *Crf* | GGGAAGTCTTGGAAATGGC | GCAACATTTCATTTCCCGAT |
| *Cypha* | GAGCTGTTTGCAGACAAAGTTC | CCCTGGCACATGAATCCTGG |
| *Esr1* | CGTGTGCAATGACTATGCCTC T | TGGTGCATTGGTTTGTAGCTGG |
| *Mest* | ACCTGCTGTCTCACGATTATGG | AGGAAAGATACCTCCATTCGAC |
| *Peg3* | AACAGCTAGGTGGCTGGTCC | TTTGCTCACACCCAAGGGCT |

***Bdnf****, brain derived neurotrophic factor;* ***Crf****, corticotrophin releasing factor;* ***Cypha****, cyclophillin A;* ***Esr1****, estrogen receptor alpha;* ***Mest****, mesoderm specific transcript;* ***Peg3****, paternally-expressed gene 3.*

Table S2: Paternal food restriction effects on litter characteristics.

|  | ET | |  | NM | |
| --- | --- | --- | --- | --- | --- |
|  | CF | FR |  | CF | FR |
| PN0 Litter Size | 9.14 ± 0.64 | 8.04 ± 0.61 |  | 11.09 ± 0.32 | 9.70 ± 0.36 |
| PN0 Litter Weight (g) | 7.17 ± 0.58 | 5.89 ± 0.56 |  | 8.80 ± 0.29 | 7.45 ± 0.31 |
| PN6 Litter Size | 6.06 ± 0.56 | 5.22 ± 0.42 |  | 6.60 ± 0.65 | 5.30 ± 0.40 |
| PN6 Litter Weight (g) | 25.12 ± 2.33 | 21.54 ± 1.55 |  | 20.33 ± 2.13 | 17.99 ± 0.19* |

*Offspring of embryo transferred (ET) and naturally mated (NM) females carrying pups of control fed (CF) or food restricted (FR) fathers, *p<0.05, different from CF offspring within the same mating condition.*

Table S3: Paternal food restriction effects on offspring body weights.

|  |  | ET | |  | NM | |
| --- | --- | --- | --- | --- | --- | --- |
|  |  | CF | FR |  | CF | FR |
| *Female Offspring* | |  |  |  |  |  |
|  | PN28 (g) | 16.13 ± 0.27 | 16.36 ± 0.23 |  | 12.98 ± 0.27 | 12.79 ± 0.22 |
|  | PN80 (g) | 22.28 ±0.28 | 21.86 ± 0.28^#^ |  | 20.35 ± 0.26 | 20.95 ± 0.19 |
| *Male Offspring* | |  |  |  |  |  |
|  | PN28 (g) | 18.43 ± 0.20 | 17.90 ± 0.28** |  | 13.88 ± 0.28 | 14.08 ± 0.22 |
|  | PN80 (g) | 27.57 ± 0.29 | 26.78 ± 0.76* |  | 26.06 ± 0.25 | 25.62 ± 0.07 |

*Offspring of embryo transferred (ET) and naturally mated (NM) females carrying pups of control fed (CF) or food restricted (FR) fathers, #p<0.10, *p<0.05, **p<0.01 different from CF offspring within the same sex and mating condition.*

Table S4: Paternal food restriction and offspring open-field behavior.

|  |  | ET | |  | NM | |
| --- | --- | --- | --- | --- | --- | --- |
|  |  | CF | FR |  | CF | FR |
| *Female Offspring* | |  |  |  |  |  |
|  | Distance travelled (m) | 23.40 ± 0.27 | 23.89 ± 0.23 |  | 24.49 ± 0.27 | 25.76 ± 0.22 |
|  | Latency to center (s) | 27.69 ± 6.39 | 31.93 ± 6.69 |  | 13.74 ± 3.77 | 18.83 ± 4.12 |
|  | Time in center (s) | 92.17 ± 5.50 | 113.20 ± 8.54 |  | 101.70 ± 10.54 | 101.67 ± 8.75 |
| *Male Offspring* | |  |  |  |  |  |
|  | Distance travelled (m) | 22.33 ± 1.01 | 22.25 ± 1.51 |  | 24.07 ± 1.11 | 23.94 ± 0.89 |
|  | Latency to center (s) | 50.40 ± 8.70 | 18.39 ± 4.39 |  | 23.10 ± 5.56 | 22.23 ± 4.47 |
|  | Time in center (s) | 107.49 ± 9.09 | 99.63 ± 12.19 |  | 109.37 ± 8.08 | 101.79 ± 7.14 |

*Offspring of embryo transferred (ET) and naturally mated (NM) females carrying pups of control fed (CF) or food restricted (FR) fathers. No significant differences.*
